# The Mydriasis-Free Handheld ERG Device and Its Utility in Clinical Practice: A Review

**DOI:** 10.3390/biomedicines14020384

**Published:** 2026-02-06

**Authors:** Marta Arias-Alvarez, Maria Sopeña-Pinilla, Diego Rodriguez-Mena, Isabel Pinilla

**Affiliations:** 1 Department of Neurophysiology, Lozano Blesa University Hospital, 50009 Zaragoza, Spain; martariasalvarez7@gmail.com (M.A.-A.); drodriguezm@salud.aragon.es (D.R.-M.); 2 Aragon Institute for Health Research (IIS Aragon), 50009 Zaragoza, Spain; mariasopenapinilla@gmail.com; 3 Department of Ophthalmology, Miguel Servet University Hospital, 50009 Zaragoza, Spain; 4 Department of Ophthalmology, Lozano Blesa University Hospital, 50009 Zaragoza, Spain; 5 Department of Surgery, University of Zaragoza, 50009 Zaragoza, Spain

**Keywords:** electroretinogram, handheld ERG, RETeval

## Abstract

**Background**: Full field electroretinography (ERG) is an essential tool for assessing retinal function and diagnosing retinal diseases. In recent years, mydriasis-free handheld ERG devices have emerged as portable, non-invasive alternatives to traditional ERG systems. Their main application has been in the screening and monitoring of diabetic retinopathy (DR), particularly in settings with limited access to standard ERG equipment and in pediatric populations where conventional testing may be difficult to perform. This review aims to evaluate the current evidence on handheld ERG devices in ocular diseases, with a focus on their reliability, diagnostic accuracy, and inherent limitations. **Methods**: A review was conducted to identify studies evaluating handheld ERG devices in diverse clinical settings, including retinal diseases, DR, pediatric populations, and conditions such as glaucoma. A comprehensive search of the Pubmed and Embase databases was performed for studies published up to December 2024. Search terms included “mydriasis free ERG”, “handheld ERG”, “portable ERG”, “RETeval”, “healthy subjects”, “retinal diseases”, “diabetic retinopathy”, “glaucoma”, and “pediatric diseases”, as well as relevant MeSH terms and synonyms. Case reports, conference abstracts, non-human studies, and letters were excluded. After screening titles and abstracts, additional studies not meeting the inclusion criteria were excluded. Of 279 records that were initially identified, 55 met the eligibility criteria and were included in the final review. Results were synthesized narratively due to heterogeneity in the study design, populations, and outcomes. Findings were organized thematically according to clinical context. **Results**: A total of 57 studies were included in the review: 19 conducted in healthy subjects, 13 in diabetic retinopathy, eight in selected retinopathies, eight in glaucoma, and 14 in pediatric cohorts. Five studies overlapped between groups due to shared populations or study designs. No meta-analysis was performed due to heterogeneity in study design and outcome measures; therefore, findings were summarized narratively across disease categories. Handheld ERG devices have been evaluated in healthy subjects, patients with DR, other retinal pathologies, glaucoma and pediatric cohorts. Evidence indicates that these devices provide a rapid, non-invasive assessment of retinal function and are particularly valuable where conventional ERG is difficult to implement and potentially well-suited for screening purposes. They show good sensitivity and reasonable specificity for detecting functional changes, making them suitable for screening purposes. However, limitations exist: reduced performance in detecting early-stage disease and cone dysfunction, risk of false positives, and variability in waveform morphology and amplitude compared with traditional ERG systems. Reproducibility challenges are noted among pediatric patients and individuals with poor fixation or unstable eye movements. These discrepancies highlight the need for establishing robust normative datasets for both healthy subjects and specific disease states. **Conclusions**: Handheld ERG devices provide a rapid, accessible and user-friendly option for retinal assessment. While not a replacement for conventional ERG, they serve as complementary tools, particularly in early disease and in contexts where standard testing is less feasible. Further research is required to refine testing protocols, improve diagnostic accuracy, and validate their application across a broader spectrum of ocular diseases.

## 1. Introduction

Full-field electroretinogram (ffERG) is essential for assessing retinal function and differentiating ocular diseases, particularly retinal pathologies and their stages. Tabletop ERG systems have long been considered as the gold standard due to their high accuracy and detailed diagnostic capabilities. More recently, handheld ERG devices, such as the RETeval^®^ (LKC Technologies, Gaithersburg, MD, USA) have gained attention for being portable, less invasive, and potentially faster alternatives. These devices use skin electrodes and pupil area-based signal adjustments without requiring mydriasis, thereby increasing ERG accessibility across a broad range of clinical settings.

The ffERG is a well-established diagnostic technique, standardized by the International Society for Clinical Electrophysiology of Vision (ISCEV) [1]. Conventional ISCEV protocols typically require corneal electrodes, pharmacologic pupil dilation, and extended dark and light adaptation periods (20 min dark, 10 min light). Full-field flash stimuli are used to evaluate both rod and cone pathways, with dark-adapted (DA) and light-adapted (LA) responses providing critical insights into photoreceptor and inner retinal function.

However, standard ERG testing can be challenging in certain populations, such as young children or individuals with limited compliance, who at times require sedation or anesthesia, which may alter ERG test results. To address these challenges, ISCEV [1] proposes an abbreviated protocol that retains Ganzfeld stimulation but shortens dark adaptation (10 min), reduces DA tests (DA 0.01 ERG and DA 10.0 ERG), and allows for testing without mydriasis. The LA 3.0 ERG and LA 3.0 30 Hz ERG are performed first, eliminating the need for a separate light adaptation phase. Other parameters, including electrode specifications, stimulus intensity, and recording settings, remain consistent with standard ISCEV protocol, regardless of pupil status. 

Other alternative approaches have been developed to enhance pediatric testing. For instance, Tony Kriss at Great Ormond Street Children’s Hospital (GOSH), London, introduced a handheld Grass photic stimulator [2], eliminating the need for sedation, mydriasis, extended dark adaptation, and corneal electrodes. While effective, this method produces lower ERG wave amplitudes [3]. Building on these innovations, the RETeval^®^ device further simplifies ERG testing by using non-invasive disposable sensor strips and eliminating pupil dilation. Although capable of performing the full range of ISCEV procedures, most research has primarily focused on cone function assessment using the ISCEV LA protocols, including the LA 3.0 cd·s·m^−2^ and the 30 Hz flicker (28.3 Hz) response, together with pupil analysis, which was initially developed for diabetic retinopathy (DR) screening [4,5].

The RETeval^®^ adjusts flash strength to maintain constant retinal illuminance (Td·s), thereby compensating for variations in pupil area (mm^2^). The device places particular emphasis on flicker ERG, which is especially sensitive to retinal vascular and ischemic dysfunction, including conditions such as DR [6,7,8]. For DA testing, the RETeval^®^ adheres to ISCEV guidelines for both rod-mediated and mixed cone/rod responses, including the DA rod-response to 0.28.

Td·s, the DA mixed cone/rod responses to 85 Td·s, the DA mixed cone–rod response with high intensity response to 280 Td·s, and the DA response to 85 Td·s oscillatory potentials (OP).

Despite its child-friendly design and non-invasive nature, pediatric compliance remains variable. Some studies report successful ERG testing in children, while others highlight challenges, particularly in younger patients or those prone to excessive blinking, photophobia, or unstable fixation. This variability is partly due to the device’s sensitivity to head position, pupil alignment, and electrode placement [9,10,11,12,13].

Normative studies have been conducted in a healthy population to establish reference datasets and to determine factors affecting its performance and reproducibility, particularly in children. In clinical settings, RETeval^®^ has been applied to a broad range of conditions, including DR, glaucoma, pediatric retinal diseases, lens opacities, drug-induced retinal toxicity, and other retinal pathologies [4,5,14,15,16,17,18,19,20,21,22].

The portable design of the RETeval^®^ system, which uses skin electrodes instead of corneal electrodes, allows for ERG recordings to be performed without pharmacological mydriasis. These characteristics, together with potentially shorter examination times, may reduce certain logistical and infrastructural requirements. Provided that DA is maintained, the device can be used in diverse environments. In this context, it has been evaluated in a variety of clinical and research settings, including screening programs and scenarios with limited access to specialized electrophysiology laboratories. Nevertheless, the acquisition of reliable recordings remains dependent on factors such as patient cooperation and access to appropriate testing conditions, which may represent limiting factors in certain populations.

The aim of this study was to systematically review and summarize the current literature on the use of this portable, mydriasis-free ERG device in both healthy individuals and patients with ocular disease. 

## 2. Materials and Methods

### 2.1. Literature Search

A comprehensive literature search was performed in the PubMed and Embase databases, with the final search being completed on 31 December 2025. No restrictions were applied regarding the publication year. The following keywords and MeSH terms, including relevant synonyms, were used: “mydriasis free ERG”, “handheld ERG”, “portable ERG” and “RETeval,” combined with terms such as “healthy subjects”, “retinal diseases”, “glaucoma”, “pediatric diseases” and “diabetic retinopathy”. Appropriate Boolean operators (AND/OR) were applied, depending on the database. Search filters were limited to studies published in English and in peer-reviewed journals. Reference lists of relevant articles were also screened to identify additional studies. No registers, websites, or gray literature sources were searched.

### 2.2. Inclusion and Exclusion Criteria

Eligible studies included original clinical or observational articles published in English that evaluated the use of mydriasis-free or portable ERG devices in ophthalmic diseases. Conference proceedings, letters, case reports, and non-human studies were excluded. Studies were grouped thematically into five categories: healthy subjects, diabetic retinopathy, selected retinopathies, glaucoma and pediatric populations.

### 2.3. Selection Process

**Figure 1:**
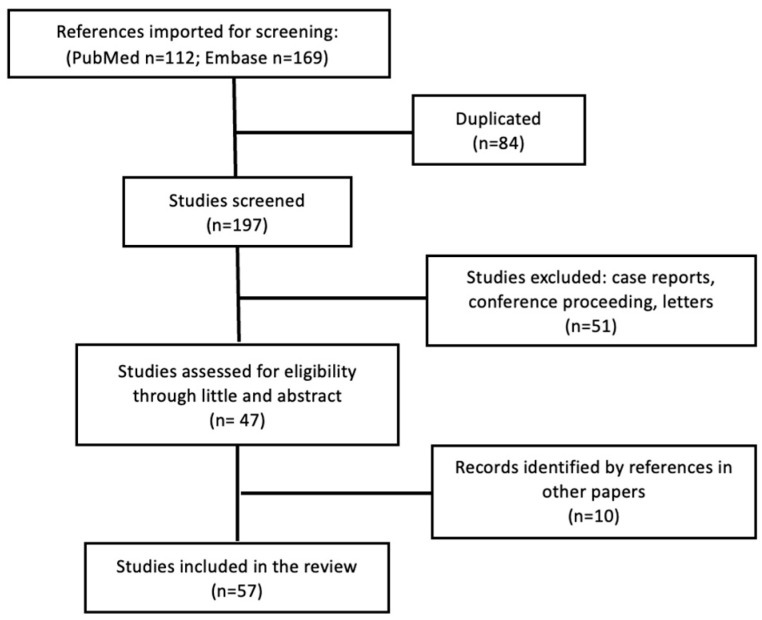
Flow diagram of the study selection process. A total of 281 records were identified (112 from PubMed, 169 from Embase). After screening and eligibility assessment, 57 studies were included in the final synthesis.

Using the defined search criteria, a total of 281 results were retrieved: 110 from PubMed and 169 from Embase. Two independent reviewers screened all titles and abstracts, with duplicates and irrelevant studies excluded at this stage. The same reviewers independently assessed full texts for eligibility, and discrepancies were resolved by consensus. No automation tools were used during screening or data selection. A total of 47 papers were selected for full-text review. Additional relevant studies identified through citation tracking were also considered. Ultimately, 57 studies met the inclusion criteria and were included in the final review. The study selection process is summarized in Figure 1.

### 2.4. Quality Assessment and Evidence Synthesis

Two independent reviewers screened and selected studies according to predefined eligibility criteria. Methodological quality was assessed using the QUADAS-2 tool for diagnostic accuracy studies and the Newcastle–Ottawa Scale (NOS) for observational studies. Extracted data included study design, population, sample size, device used, comparator tests, and outcomes of interest. Primary outcomes were diagnostic performance (sensitivity, specificity, correlation with standard ERG or clinical measures), reproducibility, and feasibility. Secondary outcomes included waveform characteristics (amplitude, implicit time (IT)) and reported limitations. 

### 2.5. Classifications

This review follows a narrative framework with a systematic search methodology. It aims to provide an evidence-based, clinically oriented synthesis of the current applications, performance, and limitations of handheld ERG systems—particularly the RETeval^®^ device—across various retinal conditions.

## 3. Results

### 3.1. Mydriasis-Free ERG in Healthy Subjects (Table 1)

#### 3.1.1. Normative Database and Reproducibility

Recent efforts have focused on establishing normative databases in healthy individuals, particularly for cone-mediated responses, and on evaluating the influence of various factors—such as pupil size, axial length (AL), sex, intraocular pressure (IOP), and lens opacities at different stages—on RETeval^®^ measurements, as well as on assessing the reproducibility of the device. Unlike conventional ERG techniques, RETeval^®^ recordings are obtained without pharmacological mydriasis, are influenced by pupil-size-dependent variations, despite illuminance and constant retinal, and use skin electrodes instead of corneal electrodes.

**Table 1:** Summary of studies assessing the performance, reproducibility, and influencing factors of portable ERG systems in healthy subjects, and cataract eyes.

Author	Condition/Disease Patients/Controls; (Mean Age (Years), [Range])	Device/Protocol	Main Findings	Limitations/Comments
Asakawa et al. [23]	50 healthy young subjects (100 eyes; 21.4 ± 0.9 y [20–24])	RETeval^®^ ISCEV and DR protocols, undilated pupils	IT more reproducible than amplitude (COV 2.5–14.6% vs. 29.8–40.8%); ICC up to 0.92	Narrow age range; lower amplitude reproducibility; variability attributed to pupil recovery and adaptation
Sommer et al. [24]	27 healthy adults (54.6 ± 8.4 y; 45–65)	RETeval^®^; ISCEV LA, flicker (28.3 Hz), PhNR; skin electrodes; natural pupils	Excellent IT reproducibility (CV~2.5–8.4% LA 3 flash/flicker; ~15.6% PhNR); amplitudes more variable (CV~18–39%), requiring ~50–89% change; detectable timing change ~1–2 ms (LA 3 flash/flicker) and ~9 ms (PhNR)	Only LA protocols; middle-aged cohort; amplitude variability limits sensitivity for longitudinal amplitude changes
Zhang et al. [25]	204 healthy children [0–18]	RETeval^®^, mydriasis-free 28.3 Hz flicker ERG	Age-dependent reference data; IT stable; amplitude ↑ with age	Possible amplitude underestimation in youngest; no diseased eyes
Inooka et al. [26]	373 healthy subjects [40–89]	RETeval^®^, 28.3 Hz flicker ERG	IT linked to age, AL, glucose; amplitude to age, platelets, creatinine	Cross-sectional; older cohort only
Stapley et al. [27]	48 myopic subjects 29.6 y [19–59] and 47 controls 27.6 y [18–55]	RETeval^®^; ISCEV 6-step dark-first protocol; skin electrodes; mydriasis	Prolonged dark-adapted ITs in myopic eyes; no significant amplitude differences under DA or LA conditions; AL positively correlated with DA ITs	High inter-subject variability; skin electrodes may limit amplitude sensitivity; anatomical confounders related to AL; limited number of high myopes
Kato et al. [28]	10 healthy subjects (33 y [25–46])	RETeval^®^, 28.3 Hz flicker, artificial vs. dilated pupils	Larger pupils → longer IT; amplitude stable; consistent with Stiles–Crawford effect	Very small sample; artificial dilation
Kato et al. [29]	150 healthy young subjects (22.8 ± 1.8 y [20–29])	RETeval^®^, 8 Td·s flicker, no background illumination	F amplitude ↑; AL and pupil area delay IT (0.1 ms/mm^2^)	Sex imbalance (2:1 M:F); non-ISCEV stimulus; limited refractive range
Kato et al. [30]	136 healthy young subjects	RETeval^®^, PhNR protocol	PhNR amplitude correlated with pRNFL; larger amplitude in females; AL, pupil, and IOP also influenced PhNR	No glaucoma eyes; limited age range
Sugawara et al. [31]	30 healthy subjects	RETeval^®^, flicker ERG at 8, 16, 32 Td·s	IT shorter in 2nd eye at 8 Td·s (esp. small pupils); sequence and pupil effects significant	Small sample; only flicker tested
Hobby et al. [32]	160 healthy subjects	RETeval^®^, 28.3 Hz flicker ERG. Natural pupils	Electrode further ↓ amplitude 40–50%; IT stable; waveform preserved	Variable ages; emphasizes need for consistent electrode placement
Kim et al. [33]	20 healthy subjects (32.6 ± 9.86 y [25–55])	LKC UTAS Bigshot (tabletop) vs. MGS-2 handheld stimulator; connected to UTAS-E3000; Mydriasis	Handheld ↓ amplitudes (DA 0.01, DA 10.0, LA 3.0, 30 Hz flicker); longer IT only for flicker; ICC 0.73–0.89	Small sample; order not randomized; amplitude diff. from retinal area/light pattern; not interchangeable; device-specific norms needed
Sachidanandam et al. [34]	57 healthy subjects (32.2 ± 14.2 y)	VERIS 5.2.2X (tabletop) vs. Ephios handheld ERG; DA 3.0 and LA 3.0 protocols	Handheld amplitudes ↓ (vs. VERIS); similar morphology; ICC for b/a = 0.66	Differences from flash intensity, retinal area, and test order; systems not interchangeable; device-specific norms needed
Liu et al. [35]	35 retinal disorders/57 healthy subjects (median 17 y [11 months–69 y]/22 y [8–65])	RETeval^®^ vs. conventional ISCEV ffERG; mostly non-mydriatic	Moderate-to-strong correlations with conventional ERG for amplitudes (*r* = 0.24–0.75) and implicit times (*r* = 0.31–0.94); strong amplitude reliability (ICC 0.79), moderate IT (ICC 0.52); *κ* = 0.82; S 1.00, SP 0.82; 87.5% diagnostic concordance	Heterogeneous cohort; incomplete tests (~50%); wide age range; mixed dilated and undilated pupils; false positives in mild dysfunction or with eye movements; requires age-adjusted normative data
Miura et al. [36]	82 cataractous/52 pseudophakic eyes	RETeval^®^, 8 Td·s flicker ERG	Cataract ↓ amplitude and ↑ IT vs. pseudophakia; effect weaker at high intensities	Subjective grading; cross-sectional; no healthy controls; cataract severity should be considered
Miura et al. [37]	32 cataract, pre/post-surgery	RETeval^®^, 28.3 Hz flicker ERG at 2, 8, and 32 Td·s	Amplitude ↑ and IT ↓ after surgery; strong stimuli reduce effect	Small sample; no mydriasis; unclear contribution of pupil vs. light scattering
Miura et al. [38]	41 cataract, grade 2 pre/post-mydriasis	RETeval^®^, 28.3 Hz flicker ERG at 2, 8, and 32 Td·s	No change pre/post-mydriasis; minimal pupil influence	Grade 2 only; sub-ISCEV intensities; limited generalizability
Kato et al. [39]	30 post-cataract surgery	RETeval^®^, 32 Td·s flicker ^r^ ERG	Transient amplitude ↑ (+31%) at 1 week, normal at 3 months; correlated with macular thickening and mild flare	Small sample; no controls; short follow-up; possible inflammatory contribution

Abbreviations: AL, axial length; b/a, b-wave to a-wave amplitude ratio; COV, coefficient of vari-ation; DA, dark-adapted; DR, diabetic retinopathy; ERG, electroretinogram; ffERG, full-field electroretinogram; F, female; ICC, intraclass correlation coefficient; IOP, intraocular pressure; ISCEV, International Society for Clinical Electrophysiology of Vision; IT, implicit time; LA, light-adapted; M, male; PhNR, photopic negative response; pRNFL, peripapillary retinal nerve fiber layer; S, sensitivity; SP, specificity; Td·s, Troland-seconds; y, years.

Asakawa et al. [23] evaluated the reproducibility and consistency of the RETeval^®^ recordings in healthy young adults to establish normative values under non-mydriatic conditions. ERGs were recorded using the ISCEV protocol, the DR assessment protocol, and pupil responses to white, red and blue light. Intra-examiner reproducibility was higher for IT than amplitudes. Inter-examiner consistency showed high-to-moderate intraclass correlation coefficients (ICC), but lower ICC values were noted for other parameters. Amplitude variability was hypothesized to result from pupil size recovery and photoreceptor adaptation following stimulation. No significant correlation was observed between pupil parameters and flicker ERG responses, although moderate correlations were found for chromatic pupil stimuli. These authors highlight its value as a screening device, particularly for detecting diseases along the visual afferent pathway. 

Sommer et al. [24] assessed the test–retest reproducibility of LA responses recorded with the handheld RETeval^®^ device, using skin electrodes and natural pupils, in healthy middle-aged adults. ITs of the LA 3.0 flash and LA flicker demonstrated excellent reproducibility with minimal intersession variability, whereas photopic negative response (PhNR) ITs were more variable. In contrast, amplitude measurements showed substantially greater variability, particularly for the LA a-wave and PhNR. These findings support the robustness of the timing parameters obtained with RETeval^®^ and skin electrodes, while highlighting the need for caution when interpreting amplitude changes in longitudinal assessments, especially in older populations.

Zhang et al. [25] evaluated mydriasis-free flicker ERGs using the RETeval^®^ system in healthy children. IT did not vary with sex, refractive error (less than −3D), or population (Chinese vs. US cohorts), whereas amplitudes increased with age and differed between cohorts. Both IT and amplitudes matured during the first decade of life. The authors noted that a suboptimal fit of the skin strip electrode in younger children may have led to amplitude underestimation in this subgroup and emphasized that age should be carefully considered when interpreting flicker ERG results.

Inooka et al. [26] assessed additional systemic and ocular factors influencing RETeval^®^ flicker recordings. After multivariate regression, IT was significantly associated with AL, age, and blood glucose levels, whereas amplitudes correlated with age, platelet count, and creatinine levels. Other parameters, such as smoking status, blood pressure, and body mass index, showed no significant effects. These findings suggest that flicker ERG outcomes are modulated not only by age but also by specific ophthalmological and hematological factors.

Stapley et al. [27] reported that the RETeval^®^ system may not detect the amplitude reductions in myopia that have been previously reported using conventional ERG systems [40,41]. In their study of non-pathological myopia, ERGs were recorded using skin electrodes, in accordance with ISCEV-standard DA and LA protocols. Compared with non-myopic controls, myopic eyes showed significantly prolonged ITs for several DA response components, while no significant differences in response amplitudes were observed under either DA or LA conditions. DA ITs showed a consistent positive association with AL across multiple ERG components. Specifically, ITs for all DA responses were positively correlated with AL, whereas LA parameters showed no significant associations. 

Kato et al. [28] investigated the effect of pupil size on flicker ERG using RETeval^®^ in 10 healthy subjects. They observed that IT increased with larger pupils, while wave amplitudes remained unchanged. This effect was attributed to the Stiles–Crawford phenomenon, which describes how cone responses depend on the angle of incident light and the effective constant retinal illuminance determined by pupil size. They concluded that adjusting flash strength alone is insufficient to ensure constant retinal illuminance, although compensation is adequate for pupil diameters below 6.5 mm.

In a subsequent study, the same group analyzed healthy young adults to identify factors influencing flicker ERG responses [29] recorded with RETeval^®^. Amplitudes were significantly higher in females, and sex was confirmed as an independent predictor of amplitude. Both AL and pupillary area were associated with delayed IT, which was consistent with prior findings using conventional ERG systems [40,42] and with the Stiles–Crawford effect [43]. The authors therefore recommend compensating for pupil-related effects: even recordings are performed without mydriasis, and it was noted that the limited refractive range of the cohort might explain why AL was not identified as an independent predictor of amplitude.

Building on Kato and colleagues’ studies on flicker ERG, Sugawara et al. [31] examined the influence of recording sequence and pupil size on flicker ERG measurements in healthy subjects. They found that ITs were shorter when recordings were obtained from the second eye in individuals with smaller pupils. This emphasizes the need to control both pupil size and recording order when performing portable ERG assessments, and it is recommended to use stronger stimuli to minimize sequence-related effects.

Beyond demographic influences, technical factors such as electrode placement also affect RETeval^®^ measurements. Hobby et al. [32] underscore the importance of consistent electrode positioning for amplitude assessment. Their results demonstrated that increasing the distance between the electrode and the eyelid significantly reduced flicker ERG amplitudes close to 50%, while IT and waveform morphology remained unaffected. These findings align with prior evidence that greater distance from the corneal apex reduces recording amplitude [44]. 

#### 3.1.2. Differences with Conventional Systems

Several studies have also focused on differences among ERG recording systems. In general, while ITs remain consistent, handheld systems typically produce lower response amplitudes than conventional tabletop systems. Reported amplitude ratios between RETeval^®^ and standard ERG systems range from 50% to 85% [45], with rod-mediated responses showing a greater reduction than cone-mediated responses [9,33].

Sachidanandam et al. [34] compared ffERG recordings between a tabletop device (VERIS 5.2.2X) and a handheld system (Ephios) in healthy participants. Under DA 3.0 ERG conditions, VERIS produced significantly larger a-wave and b-wave amplitudes, while IT differences were smaller, with only minor delays in specific responses. Despite these amplitude differences, waveform morphology was similar across devices, and the b/a ratio demonstrated good reliability. The reduced amplitudes with Ephios were attributed to factors such as lower flash intensity, smaller retinal stimulation area, device design, patient positioning, and recording sequence, emphasizing that the devices are not interchangeable and require device-specific normative databases.

Liu et al. [35] compared RETeval^®^ recordings with the standard clinical ffERG obtained according to ISCEV protocols in healthy controls and subjects with various pathologies. They concluded that the portable ERG offers a practical alternative in settings where conventional ffERG cannot be reliably implemented. Within-visit reliability of RETeval^®^ was moderate for ITs and strong for amplitudes. Agreement between the two systems was substantial (*k* = 0.82). Diagnostic performance was promising, with a sensitivity of 1.00 and a specificity of 0.82. Reliability was stronger for amplitude measurements than for ITs, and significant positive correlations were observed between corresponding ERG parameters across the two devices. Both systems successfully classified patients into categories of retinal dysfunction, with an overall concordance rate of 87.5% between devices. 

To control for confounding variables during device comparisons, Kim et al. [33] evaluated 20 healthy volunteers using two LKC stimulators, the tabletop model UTAS Bigshot and the handheld MGS-2, which were both connected to the same UTAS-E3000 system and tested under identical conditions. The overall wave morphology was similar across devices; however, the handheld stimulator produced significantly lower amplitudes in multiple protocols (DA 0.01, DA 10.0, LA 3.0, and 30 Hz flicker), while IT were comparable, with significantly longer values for the 30 Hz flicker. The waveform morphology was similar across systems, and amplitude reliability was moderate-to-high for selected scotopic responses (ICC 0.73–0.89). Both devices yielded a b-wave/a-wave > 1 ratio in DA 10.0, supporting potential clinical use in evaluating retinal ischemia and distinguishing ischemic from non-ischemic central retinal vein occlusions (CVRO). The authors attributed amplitude differences to variations in the stimulated retinal area and light distribution patterns and concluded that the systems are not interchangeable, requiring device-specific normative data.

#### 3.1.3. Influence of Lens Transparency on RETeval^®^ Measurements

The influence of cataracts on flicker ERG recordings has also been investigated. Miura et al. [36] compared responses between cataractous and pseudophakic eyes. Eyes with grade 2 or higher cataracts showed lower amplitudes and prolonged IT compared with pseudophakic eyes. The authors suggested that the effect of cataracts on IT decreases with higher stimulus intensities and recommended that cataract severity be considered when interpreting RETeval^®^ results.

In a related study, Miura et al. [37] evaluated patients before and after cataract surgery, using flicker ERGs. Following surgery, mean amplitudes increased, while ITs shortened at the two lowest intensities. These findings indicate that higher stimulus strengths mitigate the effect of cataracts on IT. 

To assess the influence of pupil size, the same group conducted a subsequent study [38] in eyes with grade 2 cataracts, comparing flicker ERGs before and after mydriasis. No significant differences in amplitude or IT were observed between dilated and undilated pupils, indicating that pupil size has a relatively minor effect on RETeval^®^ outcomes in cataractous eyes. Similarly, no significant differences were found between nuclear and cortical cataracts, implying that cataract type has little impact on light scattering in this context. These findings emphasized the importance of accounting for both cataract severity and stimulus parameters when interpreting RETeval^®^ results.

Changes following cataract surgery may also reflect intraocular inflammation in addition to lens extraction. Kato et al. [39] evaluated flicker changes after uncomplicated cataract surgery with the RETeval^®^. A transient amplitude increase was observed, peaking at 1 week postoperatively (mean increase: 31.2%) and gradually returning to baseline by 3 months, which was correlated with macular thickening and flare; IT remained stable. A secondary rise in ERG amplitude was noted around 2 months after the discontinuation of anti-inflammatory medication. Transient postoperative changes should be considered when interpreting ERGs and proposed that ERG may have potential in predicting postoperative macular edema or monitoring anti-inflammatory responses, warranting further investigation.

### 3.2. Handheld ERG Devices in DR

Building on earlier studies based on conventional ERG systems, the RETeval^®^ device was developed as a handheld tool for DR screening in patients with diabetes mellitus (DM). Its ability to operate without pharmacological mydriasis or specialized infrastructure makes it particularly well-suited for large-scale screening of DR and vision-threatening DR (VTDR) in diabetic populations (Table 2).

Maa et al. [16] were the first to describe RETeval^®^ as an efficient tool to detect VTDR. They combined ERG and pupillary response in non-dilated diabetic patients compared to standard ETDRS 7-field fundus photography. The AUC was 0.86 for VTDR. At a cutoff DR score of ≥20, the device achieved 83% sensitivity and 78% specificity for VTDR detection, with a high negative predictive value (99%) but a modest positive predictive value (15%). After excluding clinically significant macular edema, sensitivity improved to 87% and the negative predictive value to 99.2%. Testing was also faster (2.3 min for both eyes) and showed a lower failure rate (1%) compared with fundus photography (11%, *p* < 0.001). The ICC for reproducibility was 90.2%. 

In 2017, Al-Otaibi et al. [5] found that RETeval^®^ is a rapid and cost-effective screening option for DR in primary care. They compared RETeval^®^ with digital fundus photography for DR and VTDR screening. RETeval^®^ achieved excellent sensitivity for VTDR detection (95.4%) but very low specificity (17.5%), using a 20 µV amplitude cutoff, with 76% of false positives diagnosed with other posterior segment disorders. Adding sequential Amsler grid testing improved specificity up to 80.1% but reduced sensitivity to 30.1%. Despite its limitations, the test was significantly faster and more cost-efficient than fundus photography. Patient preference also favored RETeval^®^. The authors suggested that increasing the cutoff to 27 µV could improve specificity without compromising sensitivity. 

Fukuo et al. [4] evaluated the relationship between RETeval^®^ parameters and DR severity. They found no significant differences in IT or amplitude between normal subjects and DM patients without DR. In contrast, eyes with DR showed significantly longer ITs (*p* < 0.001) and lower amplitudes (*p* < 0.01) compared to both normal and subjects without DR. Flicker IT strongly correlated with DR severity (*r* = 0.55, *p* < 0.001), while amplitude showed a weaker negative correlation (*r* = −0.29, *p* = 0.001).

Using an IT cutoff of 35.6 ms, sensitivity and specificity for DR detection were 0.70 and 0.81, respectively, with an AUC of 0.84. Diagnostic accuracy was superior for IT compared with amplitude (AUC 0.84 vs. 0.67). For identifying eyes requiring treatment, IT also outperformed amplitude (AUC 0.89 vs. 0.66). With an IT cutoff of 36.4 ms, both sensitivity and specificity reached 0.85. The authors concluded that flicker IT is a robust screening parameter for DR and treatment-requiring DR, but emphasized the need to optimize stimulus parameters and analyze full raw data to maximize diagnostic accuracy.

Değirmenci et al. [17] found that RETeval^®^ is a more effective screening tool for VTDR than structural OCT parameters. They studied diabetic patients with varying DR stages and measured macular RNFL thickness with optical coherence tomography (OCT) as a marker of diabetic neurodegeneration. ffERG were recorded using the DR assessment protocol, which integrates IT, amplitude, age and pupil response. Higher DR severity was associated with higher risk scores, but macular RNFL thickness and central macular thickness (CMT) showed no correlation with DR severity. At a cutoff of 20, RETeval^®^ achieved 100% sensitivity but only 67% specificity. Adjusting the cutoff to 22 optimized both sensitivity (92%) and specificity (92%). 

Zeng and colleagues looked for the best cut-off in type 2 DM patients with RETeval^®^ [19]. The DR protocol differed significantly among all five groups (no DR, mild, moderate and severe NPDR and proliferative DR (PDR)). The AUC for overall DR detection was 0.867, with optimal sensitivity and specificity of 80.2% and 81.7%, at a cutoff of 20.75. For VTDR detection, performance improved substantially (AUC 0.965), with a cutoff of 23.05, yielding 94.6% sensitivity and 88.8% specificity. RETeval^®^ thus distinguished DR from no DR and VTDR from non-VTDR, with better accuracy for VTDR. The authors noted that cutoff values may require population-specific adjustments, considering factors such as ethnicity and glycemic control.

In another investigation, Zeng’s group assessed early neurovascular impairments in type 2 DM patients without clinically detectable DR, using OCT, OCT angiography (OCTA), and flicker ERG [46]. Diabetic eyes showed reduced parafoveal and perifoveal vessel density (VD) in both superficial (SCP) and deep capillary plexuses (DCP). While ganglion cell complex (GCC) thickness was preserved, both pRNFL and the radial peripapillary capillary plexus VD were reduced. Functionally, DM patients exhibited delayed ITs, decreased amplitudes, higher DR scores, and smaller pupil area ratios. Increased ITs correlated with decreased SCP VD, while IT delay at 32 Td·s also correlated with a reduced perifoveal DCP VD. Regression analysis showed that delayed IT correlated with the HbA1c and parafoveal SCP VD reduction. The authors concluded that even in the absence of clinically visible DR, diabetic eyes demonstrate microvascular and functional impairments, most notably IT delay, which may serve as an early biomarker of preclinical DR.

In a related study of type 2 DM patients, the same group [18] examined the relationship between OCTA microvascular parameters and RETeval^®^ function. Reduced amplitudes, delayed IT, and decreased VD were present, even in patients without clinically detectable DR, worsening with increasing DR severity. Parafoveal VD in both SCP and DCP along with age correlated strongly with IT changes. In NPDR, reduced amplitudes were linked to decreased parafoveal VD, while IT delay was associated with both age and VD loss. These findings support parafoveal VD and flicker ERG as sensitive markers of early functional impairment, with flicker amplitude becoming increasingly valuable in monitoring DR progression, particularly in moderate-to-severe NPDR.

Brigell et al. [47] examined the predictive value of RETeval^®^ for DR progression. Patients with a DR risk score ≥ 23.5 were 11 times more likely to require ocular intervention. ETDRS-DR severity ≥ 53 conferred a 3.5-fold increased risk, while coexisting DME raised risk 4.7-fold. When both structural and functional parameters were positive, the risk rose 15-fold. The authors highlighted the value of combining structural and functional assessments to refine the referral criteria and improve prognostic accuracy.

Deng et al. [20] explored a population-specific DR screening model for Chinese type 2 DM patients. With increasing DR severity, ERG amplitudes decreased, ITs increased, and pupil responses worsened. For DR detection, the AUC was 0.881; the optimal cutoff of 22.95 achieved 74.3% sensitivity and 90.6% specificity. Using the default threshold of 20 increased sensitivity (87.6%) but reduced specificity (48.8%). For VTDR detection, performance was excellent (AUC 0.972), with a threshold of 26.45 yielding 95.7% sensitivity and 93.5% specificity. Predictive values improved further when combined with clinical variables such as BCVA, DM duration, and HbA1c.

While DR scores did not differ significantly between controls and DM patients without DR, flicker amplitudes at 16 and 32 Td·s were reduced. The authors concluded that RETeval^®^ is especially valuable for diagnosing VTDR rather than early DR, consistent with findings from Fukuo and Zeng [4,19]. Their integrated algorithm demonstrated improved sensitivity compared to earlier reports [16,19].

Kirthi et al. [21] examined early neurovascular dysfunction in prediabetes, using handheld ERG, OCTA, corneal confocal microcopy, and electrochemical skin conductance in prediabetics and type 2 DM [22]. They observed a reduction in flicker amplitudes. Parafoveal VD in both SCP and DCP was reduced in prediabetes and type 2 DM compared to the controls. Corneal nerves and electrochemical skin conductance were preserved in prediabetic subjects but impaired DM patients. 

HbA1c showed a negative correlation with 32 Td·s peak amplitude and a positive correlation with ITs for both flicker stimuli. Based on these findings, the authors proposed that 32 Td·s flicker provides greater discriminatory power than 16 Td·s for detecting early retinal dysfunction. Their results demonstrate that both retinal function (measured by ERG) and vascular health (assessed by OCTA) are already compromised in prediabetic subjects, supporting handheld ERG as a sensitive tool for early detection of diabetes-related neurovascular disease. 

Weerasinghe et al. [14] conducted a larger study integrating non-mydriatic fundus photography with RETeval^®^ to assess DR prevalence and screening accuracy in a high-risk diabetic population. Mydriatic retinal images were used as the reference standard. Non-mydriatic photography showed a high failure rate of 56.7% compared to 7.6% for the mydriatic images; its specificity and sensitivity for DR detection were 20.2 and 99.5%, respectively. RETeval^®^ demonstrated more balanced results, yielding a sensitivity of 72.5% and a specificity of 70.1% for DR detection, though with a 15% failure rate. An optimal DR cutoff score was 22, with a positive predictive value of 64.9% and negative predictive value of 77.0%. 

Multivariable regression identified strong predictors of DR, including a diabetes duration of 5–24 years, HbA1c levels ≥ 7%, reduced estimated glomerular filtration rate (eGFR) (≤29 mL/min/1.73 m^2^), pupil size < 4 mm (likely due to diabetic autonomic neuropathy), and a DR score ≥ 22. The authors suggested that portable mydriatic systems could be particularly valuable in high-risk DM populations or undiagnosed disease. While a DR score ≥ 22 was a strong predictor of DR, they cautioned against using RETeval^®^ as a standalone screening device due to its moderate specificity and recommended further cost-effectiveness studies.

Zhuang et al. [15] investigated the combined use of RETeval^®^, ultrawide field (UWF) imaging, and OCTA to assess both functional and neurovascular changes in patients with peripheral DR lesions. RETeval^®^ detected functional abnormalities associated with peripheral lesions, particularly at higher stimulus intensities (32 Td·s), including longer IT, reduced amplitudes, and smaller pupil area ratio. The DR score was also significantly different between groups. After adjustment, IT changes remained significantly associated with the presence of peripheral lesions. The authors concluded that retinal function is more impaired in DM patients with peripheral lesions and that combining UWF imaging with functional testing enhances DR severity assessment. The combination of UWF imaging and functional tests improves the evaluation of DR severity.

Arias-Alvarez et al. [48] conducted a direct comparison of retinal function measured with a conventional ERG system and the portable RETeval^®^ device. Significant functional abnormalities were detected in diabetic patients despite the absence of visible DR. Both systems identified changes under scotopic conditions—particularly delayed IT and reduced oscillatory potential (OP) amplitudes—suggesting early neurodegeneration. Only the conventional system detected photopic abnormalities, such as reduced b-wave and flicker amplitudes.

Regarding the DR assessment protocol, significant differences between patients and controls were only observed at the 16 Td·s 28.3 Hz flicker (both IT and amplitude), but not at 32 Td·s or in the DR risk score. Despite these discrepancies, the two devices showed a good correlation across most parameters. Both primarily revealed scotopic alterations, with OPs proving especially sensitive to early dysfunction, even before fundus lesions appeared. 

### 3.3. Handheld ERG Devices in Selected Retinopathies

Although RETeval^®^ has been primarily used in DR, its application extends to other retinal conditions (Table 3).

Yasuda et al. [49] supported the role of repeated flicker ERG recordings as a quantitative biomarker for ischemia in patients with macular edema, secondary to CRVO undergoing intravitreal ranibizumab injections [56]. Before treatment, ITs were significantly prolonged in CRVO eyes compared to fellow eyes. RETeval^®^ IT was slightly longer than that recorded with the conventional system, with no statistically significant differences and a strong correlation between systems (*r* = 0.89, *p* < 0.001). Amplitudes also correlated significantly (*r* = 0.66, *p* < 0.008). One month after treatment, IT shortened significantly in affected eyes without changes in amplitude, suggesting recovery of retinal function and/or an improvement in the retinal ischemia or a decrease in retina edema. 

Terauchi et al. [50] demonstrated RETeval^®^’s sensitivity to subtle bilateral electrophysiological changes shortly after anti-VEGF treatment. After the injection, flicker amplitudes remained unchanged, but ITs were significantly prolonged in injected eyes, except in DME eyes within the first 2 hours. Interestingly, ITs in contralateral eyes were also prolonged, reaching significance at 2–24 hours: a finding that was absent in Yasuda’s study, where IT improved after one month [49]. Drug-specific effects were also noted. The authors attributed these early IT changes to transient procedure-related factors such as IOP elevation or systemic drug exposure, rather than therapeutic effects. 

Miyata et al. [51] used RETeval^®^ to explore the clinical significance of supernormal flicker ERG amplitudes in CRVO. CRVO eyes showed prolonged ITs compared to fellow eyes. In 20.8% of cases, amplitudes were paradoxically supernormal (≥117% of fellow eye), corresponding to non-ischemic CRVO, and better VA at 12 months. After anti-VEGF treatment, amplitudes in supernormal eyes decreased significantly, whereas no changes were observed in ischemic CRVO or non-ischemic cases without supernormal responses. ITs remained stable across groups. The authors proposed that supernormal amplitudes may reflect elevated intraretinal VEGF levels and mild ischemia.

Handheld ffERG systems have also been tested for inherited retinal dystrophies. Nakamura et al. [52] evaluated the feasibility of RETeval^®^ flicker recordings without mydriasis in confirmed cone dysfunction, as determined by conventional ERG systems. RETeval^®^ effectively differentiated the eyes with severe cone dysfunction, showing significantly reduced amplitudes and prolonged IT. RETeval^®^ amplitudes correlated significantly with conventional ERG, but ITs did not, likely reflecting differences in recording conditions. 

They concluded that RETeval^®^ is promising for detecting severe cone dysfunction, but discrepancies in waveform and timing compared to tabletop ERG limit its use for mild cases. Standardized protocols are needed before adopting RETeval^®^ for inherited retinal dystrophy screening.

You et al. [45] compared the handheld RETeval^®^ with the conventional Espion systems in healthy subjects and in patients with retinal disorders affecting the ON- and OFF-pathways, including complete congenital stationary night blindness (CSNB, ON-pathway dysfunction) and congenital post-receptoral cone pathway anomaly (CPCPA, OFF-pathway dysfunction). Although RETeval^®^ produced reliable and reproducible responses, notable discrepancies in OFF-pathway stimulation were observed when compared with conventional tabletop systems. In healthy eyes, RETeval^®^ recordings showed reduced b-wave amplitudes, a slower return to baseline, and absence of the i-wave under photopic conditions: features suggesting a weaker stimulation of the OFF-pathway. 

Intersystem discrepancies were largest in CSNB (an ON-pathway disorders), with no statistically significant differences between systems in the CPCPA patients. Although RETeval^®^ complied with ISCEV standards and demonstrated good reproducibility and a COV comparable to that of the Espion system (22.5 vs. 23.4, respectively), the persistent amplitude and waveform differences, particularly concerning the OFF-pathway, highlight the need for careful interpretation of its findings.

Han et al. [53] found that flicker amplitude assessed with RETeval^®^ has prognostic value in eyes with vitreous hemorrhage. Despite media opacity, flicker ERGs could be recorded in all eyes. Amplitude ratios were markedly reduced in eyes with RRD. Using a cutoff of 0.14, the AUC for rhegmatogenous retinal detachment (RRD) detection was 0.977, with 100% sensitivity and 95.4% specificity. 

Shibuya et al. [54] suggested that RETeval^®^ can provide useful postoperative functional data, even in partially gas-filled eyes after pars plana vitrectomy (PPV) for RRD. Reliable flicker recordings were achievable, even in eyes with half-filled gas tamponade. Amplitudes recorded at both postoperative timepoints correlated positively, indicating that early post-PPV recordings could predict later outcomes. Recovery, reflected by amplitude ratios, was greater in eyes with limited detachment (≤2 quadrants) and in those treated within 7 days of symptom onset. 

RETeval^®^ has also been tested in posterior uveitis. Waldie et al. [55] evaluated its ability to assess retinal function in birdshot chorioretinopathy. RETeval^®^ and the conventional results correlated strongly (*r* > 0.75, *p* < 0.01) under both used protocols. Amplitudes were consistently lower with RETeval^®^—approximately threefold lower than conventional ERG. Amplitude reductions also correlated negatively with disease duration. In advanced cases, flicker responses were occasionally undetectable with RETeval^®^, but remained measurable with conventional ERG. Bland–Altman analysis revealed small peak time differences. Overall, RETeval^®^ proved to be feasible and rapid for monitoring cone function in panretinal disease, but differences in amplitude and waveform—particularly under photopic conditions—underscore the importance of protocol choice and cautious interpretation.

### 3.4. Handheld ERG Devices in Glaucoma (Table 4)

ERG has demonstrated significant potential in diagnosing and monitoring glaucoma by assessing functional changes through the PhNR (Table 4).

**Table 4:** Summary of studies assessing the PhNR using the RETeval^®^ system in healthy subjects and in patients with glaucoma.

Author	Condition/Disease Patients/Controls	Device/Protocol	Main Findings	Limitations/Comments
Hidaka et al. [11]	Primary open-angle glaucoma (90 mild and 76 moderate–advanced)	RETeval^®^, PhNR; six PhNR and two ERG parameters (a-, b-waves); correlation with MD and cpRNFL	Flicker ERG recordable despite All PhNR parameters except IT were reduced in glaucoma; BT and P_72_ amplitudes decreased even in mild POAG; strongest correlations with MD and cpRNFL in moderate–advanced disease; BT most diagnostic (AUC 0.947)	Lower sensitivity in early glaucoma; localized ganglion damage limits early detection; cross-sectional design
Kato et al. [30]	136 healthy young subjects	RETeval^®^, PhNR protocol; red flash (38 Td·s) on blue background (380 Td), 3.4 Hz	PhNR amplitude (P_72_, P_min_) and pRNFL thickness significantly correlated; larger a-wave, b-wave and PhNR amplitudes in females; pRNFL emerged as independent predictor of all PhNR indices	No glaucoma eyes; weak correlations due to narrow pRNFL range; limited signal-to-noise with skin electrodes; young, healthy cohort; sex and age effects on amplitude
Wu et al. [57]	20 healthy subjects (40 eyes)	RETeval^®^, PhNR; red flashes (4 ms, 58 Td·s) on blue background (10 cd·m^−2^), 3.43 Hz, 200 sweeps	PT/B ratio showed best repeatability (%CoR = 30 ± 4%); consistent within/between examiners; repeatability improved with more sweeps	Small sample; only healthy eyes; findings limited to repeatability, not pathology
Tang et al. [58]	20 glaucoma/18 controls	RETeval^®^, PhNR; 50 red flashes (1.6 cd·s∙m^−2^) on blue background (10 cd·m^−2^), 2 Hz	Baseline detrending with third-degree polynomial improved PhNR test–retest repeatability (CoR ± 44%) without loss of diagnostic accuracy (AUC 0.74 vs. 0.75 with standard filter)	Small sample; focused on signal processing, not clinical validation; higher-order detrending may alter waveform morphology.
Tang et al. [59]	21 glaucoma/10 glaucoma suspects/36 controls	RETeval^®^, PhNR; 100 red flashes (≤4 ms, 1.7 cd·s·m^−2^) on blue background (10 cd∙m^−2^)	Sensor strips showed good repeatability and correlation of PhNR amplitude, IT, and PhNR/b ratio with mean deviation (MD), but yielded ⅓ DTL amplitudes, lower SNR, and longer ITs	Lower SNR and amplitudes with sensor strips; less reliable in low-signal eyes; DTL preferred for precision.
Kita et al. [60]	Primary open-angle glaucoma (62 early and 39 moderate–advanced)	RETeval^®^, PhNR; red flash on blue background; analysis of P_72_, W-ratio, P-ratio, and minimum amplitude	RETeval^®^ responses reproducible PhNR parameters correlated with MD and cpRNFL; strongest with P_72_ amplitude. RETeval^®^ distinguished moderate–advanced glaucoma (AUROC 0.92–0.96 for W-ratio), comparable to OCT; less sensitive for early disease	Controls younger than patients; single-ethnicity cohort; limited sensitivity in early glaucoma; no validation in high myopia or media opacities
Bekollari et al. [61]	59 glaucoma eyes/63 controls	RETeval^®^, PhNR; red flash (38 Td·s) on blue background (380 Td at 3.4 Hz; 400 sweeps; correlation with OCT	↓ Minimum PhNR amplitude in glaucoma before marked visual field loss; cpRNFL correlated with b-wave and W-ratio; supports combined structural–functional assessment for early detection	Small sample; single ethnicity (Caucasian); inter-study variability due to population and OCT-RETeval^®^ differences
Bekollari et al. [62]	73 glaucoma (various stages)/78 control eyes	RETeval^®^; PhNR parameters analyzed with machine learning classifiers (SVM, etc.); comparison with OCT-based models	RETeval^®^ with SVM achieved 93% accuracy (S 89.9%, SP 95.2%, AUC 0.911) using 4 parameters; outperformed OCT (81.1% accuracy, +14.7%); consistent across eye- and sex-based classifications	Retrospective; single-ethnicity cohort; requires prospective validation and optimized feature selection

Abbreviations: AUC, area under the curve; AUROC, area under the receiver operating characteristic curve; BT, baseline-to-trough amplitude; CoR, coefficient of repeatability; cpRNFL, circumpapillary retinal nerve fiber layer; ERG, electroretinogram; IT, implicit time; MD, mean deviation; OCT, optical coherence tomography; PhNR, photopic negative response; POAG, primary open-angle glaucoma; P_72_, amplitude measured 72 ms after b-wave peak; pRNFL, peripapillary retinal nerve fiber layer; PT/B, peak-to-trough/b-wave ratio; S, sensitivity; SNR, signal-to-noise ratio; SP, specificity; SVM, support vector machine; Td·s, Troland-seconds; and W-ratio, (b-wave−PhNR trough)/b-wave.

The PhNR is typically reduced in glaucoma and has been correlated with thinning of the pRNFL. Machida et al. [63] reported that PhNR amplitudes were decreased in glaucomatous eyes and showed linear correlations with pRNFL thickness and optic disk parameters, supporting its role as a functional biomarker of retinal ganglion cell loss.

Kato et al. [30] evaluated factors influencing the PhNR recorded with the RETeval^®^ system and its relationship with pRNFL thickness in healthy subjects. They found that PhNR amplitudes (P_72_ and P_min_), as well as a-wave and b-wave amplitudes, were significantly larger in females. PhNR amplitude and pRNFL thickness were significantly correlated across all indices (P_72_, P_min_, P-ratio, and W-ratio). They also noted that AL, pupil diameter, and IOP influenced PhNR measurements; however, in a multivariable analysis, pRNFL thickness emerged as the strongest independent factor that was significantly associated with all PhNR indices, supporting its role as a surrogate marker of ganglion cell function. These findings suggest that PhNR amplitude and pRNFL are correlated, even in healthy eyes, and that RETeval^®^ could be a useful screening tool for glaucoma, though interpretation should account for age, electrode type, and sex.

Wu et al. [57] investigated the repeatability of PhNR measurements in healthy subjects using RETeval^®^ with skin electrodes. Among five PhNR amplitude metrics tested, the PT/B ratio (defined as the amplitude from the PhNR trough to the b-wave peak, divided by the b-wave amplitude from the a-wave trough to the b-wave peak) showed the lowest coefficient of repeatability. Repeatability of the PT/B ratio was consistent within and between examiners, and across sessions. The authors concluded that the PT/B ratio is the most robust PhNR metric for longitudinal monitoring of retinal function in glaucoma and other neuroretinal diseases.

Tang et al. [58] evaluated different signal processing methods to improve test–retest reliability in glaucoma and found that baseline detrending, particularly with a third-degree polynomial model applied across the entire waveform, provided the best PhNR test–retest repeatability without compromising diagnostic accuracy. This approach effectively reduced baseline fluctuations that obscure the PhNR trough. 

Electrode type also significantly influences PhNR recordings, as signal quality and reproducibility may vary. In a follow-up study, Tang et al. [59] compared sensor strips and DTL electrodes. While sensor strips demonstrated good intersession repeatability and correlations of PhNR amplitude, IT, and PhNR/B ratio with mean visual field deviation (MD), they yielded significantly lower PhNR amplitudes and had poorer signal-to-noise ratios. Sensor strips also tended to produce longer ITs. These limitations were particularly evident in eyes with attenuated PhNR or greater background noise. Consequently, DTL electrodes remain preferable in cases requiring greater precision and waveform fidelity. Nevertheless, as Wu et al. [57] demonstrated, PT/B ratios obtained with sensor strips were reproducible and unaffected by electrode placement in healthy subjects, supporting their utility in less demanding clinical settings.

Further research has assessed the ability of RETeval^®^ to gauge glaucoma severity by correlating PhNR parameters with structural and functional measures. Kita et al. [60] found that several RETeval^®^ parameters (including PhNR amplitude, W-ratio, P-ratio, and P_72_ PhNR amplitude) correlated significantly with visual field MD in moderate-to-advanced POAG and with circumpapillary RNFL (cpRNFL) thickness in all glaucoma subjects. Among these, P_72_ amplitude showed the strongest association with both MD and OCT metrics. 

RETeval^®^ was particularly effective in distinguishing moderate-to-advanced glaucoma from healthy eyes that was comparable to inner macular thickness on OCT. For early glaucoma detection, however, RETeval^®^ was less sensitive than OCT: the best performing PhNR parameter (minimum amplitude, AUROC = 0.905) was still inferior to average cpRNFL and macular ganglion cell–inner plexiform layer (mGCIPL) thickness (AUROC ≥ 0.927 and 0.938, respectively). 

Building on these findings, Hidaka et al. [11] assessed six PhNR parameters and two ERG parameters (a- and b-wave amplitudes) in relation to visual field MD and cpRNFL thickness. All PhNR parameters except IT were significantly altered in glaucoma compared with controls, with the base-to-trough (BT) and P_72_ amplitudes reduced, even in mild POAG. Correlations between PhNR parameters and MD or cpRNFL thickness were stronger in moderate-to-advanced glaucoma, with BT emerging as the most diagnostically useful parameter. These results corroborate prior evidence regarding that PhNR provides diagnostic value comparable to OCT, particularly by mitigating the floor effect observed in visual field testing in advanced POAG. However, its sensitivity for early detection remains limited. 

Subsequently, Bekollari et al. [61] investigated the relationship between structural (OCT) and functional (RETeval^®^ ERG) changes in early glaucoma. Significant reductions in minimum PhNR amplitude were observed in glaucoma patients, indicating functional impairment prior to marked visual field loss. In addition, the cpRNFL thickness correlated significantly with the b-wave and W-ratio PhNR parameters in glaucomatous eyes, supporting the value of integrating functional and structural metrics for early detection. 

In a follow-up study, Bekollari et al. [62] explored whether machine learning (ML) approaches could enhance the diagnostic performance of RETeval^®^ for glaucoma classification. Three ML classifiers were applied to compare RETeval^®^-based classification with OCT-based classification. RETeval^®^ achieved the highest accuracy with the Support Vector Machine (SVM) classifier, with 93% accuracy (sensitivity 89.9%; specificity 95.2%) when using four parameters. In comparison, OCT-based classification achieved 81.1% accuracy, representing a 14.7% improvement in favor of RETeval. RETeval^®^ also demonstrated superior accuracy for eye-specific and gender-based classifications. 

### 3.5. Handheld ERG Devices in Pediatric Subjects

Diagnosing retinal disorders in children presents significant challenges due to limited cooperation and the discomfort associated with conventional ERG methods, which require contact lens electrodes and mydriasis. Early detection of ocular disease is crucial, particularly for inherited retinal dystrophies, and is becoming increasingly important with the emergence of gene therapies. When cooperation is minimal or absent, general anesthesia may be necessary to obtain reliable ERG recordings.

The RETeval^®^ system addresses several limitations of conventional ERG by using skin electrodes and eliminating the need for mydriasis, thereby increasing comfort and reducing the need for sedation or anesthesia. However, the system has certain drawbacks. Several studies have compared RETeval^®^ with conventional ERG in children, establishing normative values in both healthy and diseased cohorts and assessing its performance in pathological conditions (Table 5). 

Soekamto et al. [64] established normative values for healthy children using RETeval^®^. A moderate positive correlation was observed between age and both OPs and DA 0.01 amplitude (*r* = 0.59, *p* = 0.006 for both). A strong positive correlation was found between age and cone a-wave IT (*r* = 0.67, *p* = 0.001). No significant correlation was found between age and the remaining ERG parameters. The authors emphasized the importance of proper electrode placement to minimize variability and amplitude reduction. Normative mean amplitudes and ITs for each ISCEV response were provided by age group. They concluded that RETeval^®^ allows for reliable ffERG testing in children without anesthesia and that age correlates with several ERG parameters.

Chan et al. [65] developed a reference dataset for photopic ERG with RETeval^®^. Age was weakly but significantly positively correlated with b-wave amplitude and 30 Hz flicker amplitude, but negatively correlated with their ITs, which was consistent with ongoing retinal maturation in early childhood. No significant sex differences were observed. AL was negatively associated with a-wave amplitude (*r* = –0.13, *p* < 0.01) and 30 Hz flicker IT (*r* = −0.10, *p* < 0.04), but positively associated with a-wave IT (*r* = 0.12, *p* ≤ 0.01), b-wave amplitude (*r* = 0.15, *p* ≤ 0.01), and 30 Hz flicker amplitude (*r* = 0.14, *p* < 0.01). No correlations were found between the SER and ERG responses. These findings highlight retinal maturation processes during preschool years, as reflected in age and AL-related ERG changes.

Several retinal disorders, including inherited retinal dystrophies, have been assessed using RETeval^®^ and a conventional ERG system in pediatric populations [9,35,52], as previously detailed in the corresponding sections. 

Eye movement artifacts remain a significant challenge when interpreting RETeval^®^ recordings, particularly in patients with nystagmus and retinal dystrophies. In Grace et al.’s study [12], nystagmus patients with retinal dystrophies exhibited significantly lower amplitudes and longer ITs. The authors suggested that poor fixation may compromise signal averaging, leading to artifacts even with cooperation and pupillary dilation. Amplitude proved highly discriminative, effectively differentiating nystagmus patients with and without retinal dystrophy. A cut-off <5 µV achieved 93% sensitivity and 94.7% specificity for identifying retinal disorders, while a stricter threshold of 2.54 µV yielded 93% sensitivity and 100% specificity. Results below 5 µV combined with IT > 33 ms warranted further exploration, while amplitudes ≥2.54 µV were associated with a 90% probability of normal cone function. Good interocular agreement was noted in healthy children and in nystagmus patients without dystrophies. Age had no measurable effect, although infants were not included. The authors concluded that RETeval^®^ flicker ERG is a feasible, fast, and effective screening tool for differentiating nystagmus patients with retinal dystrophy. 

Osigian et al. [66] found that RETeval^®^ flicker ERG could serve as a screening test for cone dysfunction. RETeval^®^ consistently produced smaller amplitudes than conventional ERG before and after general anesthesia, which was likely due to the use of skin electrodes, but strong correlations were observed for amplitude across the methods, and moderate correlations for ITs. Unexpectedly, RETeval^®^ amplitudes were smaller before than after anesthesia. Using a 5 µV cut-off, RETeval^®^ achieved a positive predictive value of 85% and a negative predictive value of 90% for detecting retinal dysfunction. Abnormal or inconclusive results would still require confirmation with standard ERG. 

Carter et al. [9] compared the handheld RETeval^®^ system with standard ERG (Espion 300) in patients with inherited retinal dystrophies. In adults, amplitudes were consistently lower with RETeval^®^, except for the flicker response, which showed higher amplitudes, while IT differences were smaller. In pediatric patients, both devices demonstrated high diagnostic agreement (*κ* = 0.80). RETeval^®^ achieved 100% sensitivity and 91% specificity for detecting abnormal responses. The authors noted that absolute agreement between devices was lacking, emphasizing the importance of device-specific normative data. Most adults preferred the tabletop system, due to shorter testing times and greater comfort. They suggested that RETeval^®^ could serve as a practical triaging tool, particularly in pediatric populations. 

Haseoka et al. [67] tested RETeval^®^ in diagnosing blue cone monochromacy (BCM). The rod and maximal responses were normal, while cone and flicker responses were markedly reduced or absent. Positive S-cone responses were detected at 30–40 ms, with the higher stimulus intensity in all patients supporting the BCM diagnosis. The study demonstrated RETeval^®^’s feasibility for minimally invasive diagnosis in children, though it was limited by the absence of normative S-cone ERG data and ƒ control subjects. 

Ji et al. [13] assessed RETeval^®^ feasibility and intra-visit reliability in children receiving vigabatrin, a drug with known retinal toxicity. Results were compared with sedated ERG. RETeval^®^ showed strong intra-visit reliability for amplitudes and moderate for IT. Amplitudes correlated strongly with Espion ERG (*ω^2^* = 0.71), but ITs did not. While amplitudes were smaller with RETeval^®^, likely due to differences in electrode type, the waveforms were similar. Despite challenges in very young children, the authors concluded that RETeval^®^ may be a viable alternative for monitoring vigabatrin-treated patients. 

Nagarajan et al. [68] found that RETeval^®^ 30 Hz flicker is a useful tool for monitoring vigabatrin’s retinal toxicity. Flicker amplitudes were significantly reduced after treatment. At 6 months of treatment, 72.7% of children showed retinal toxicity, and no recovery was observed after drug cessation. Toxicity was associated with treatment duration, rather than cumulative dose, and was independent on treatment onset age. 

Several groups have applied portable flicker ERG to assess retinal development in premature infants. Tekavčič Pompe et al. [69] compared term-born children with preterm children with or without retinopathy of prematurity (ROP). ITs were significantly prolonged in preterm children, especially those with ROP (*p* < 0.0001). These findings suggest delayed cone pathway maturation, which was possibly related to impaired OFF-bipolar cell development. Flicker amplitudes did not differ significantly between groups. The authors proposed that portable ERG may be useful for screening retinal function in preterm children, although it lacks the diagnostic sensitivity and specificity of Ganzfeld ERG. 

Based on evidence that RETeval^®^ can record flicker ERG in pediatric populations without mydriasis and in test preterm infants, Hanson et al. [70] validated the feasibility of 28.3 Hz flicker ERG recordings in newborns, using a customized protocol as a preliminary step toward assessing preterm infants at risk of ROP. Reproducible and reliable flicker ERGs were obtained in all infants, with the highest stimulus intensities producing the most consistent responses. In most cases, ERGs were also detectable at lower intensities and remained measurable even at LA 3 cd·s·m^−2^. Amplitudes increased with stronger stimuli, while ITs decreased slightly. Postprandial testing was the most favorable time for recordings. The authors concluded that RETeval^®^ offers a fast, noninvasive method for early assessment of cone function in sleeping neonates with closed eyelids and without mydriasis.

Taner et al. [71] evaluated preterm-born children using RETeval^®^ through closed eyelids. The most reproductible ERGs were obtained with the 30 cd·s·m^−2^ stimulus. Recordings were generally well-tolerated, though technical challenges arose from small facial structures and fragile skin. Amplitudes increased with stimulus strength, while ITs remained unchanged. Although ERGs were recorded in 88% of moderately preterm and 85% of extremely preterm children with the optimal stimulus, recordings were less reliable than in term-born infants. The authors emphasized the need for improved electrode designs tailored to premature infants, recommended larger cohorts including more children with ROP, and proposed longitudinal studies to better characterize cone system maturation in this population.

Expanding the use of portable ERG systems in pediatric retinal disease, Sachidanandam et al. [72] studied patients with retinoblastoma (RB), using the Ephios hand-held portable ERG system. Patients were classified according to the International Intraocular Retinoblastoma Classification (IIRC). Both a- and b-wave amplitudes were reduced across all RB subgroups, with statistically significant decreases in b-wave amplitudes in all groups except the 30 Hz flicker response. Greater amplitude reduction was seen in eyes that were classified as groups C and D than in groups A and B. ITs were significantly prolonged in groups A to C compared with the controls, except for the single-flash rod response. Tumor activity (active vs. regressed) did not significantly influence ERG parameters in groups A and B. In one longitudinal case, serial ERGs demonstrated progressive improvement in b-wave amplitudes during chemotherapy, supporting ERG’s value in monitoring functional recovery. The authors noted that the disproportionate reduction in b-waves compared with a-waves suggests a stronger impact on bipolar cells, either from the tumor or from chemotherapy. They concluded that ERG amplitudes and ITs are significantly altered in RB compared with the controls, and that b-wave assessment in particular offers a valuable adjunct for evaluating the retinal function and treatment response in RB.

## 4. Discussion

Handheld ERG devices, particularly the RETeval^®^ system, have gained increasing use in assessing retinal function across a wide spectrum of conditions, including DR, selected retinopathies, glaucoma, and in the pediatric population. These devices provide a portable, accessible, and non-invasive alternative to conventional ERG systems, which typically require pharmacological pupil dilation, corneal electrodes, specialized equipment, and trained specialists. Several studies have highlighted the advantages of handheld ERG, particularly their ability to enable rapid screening in diverse ophthalmic diseases, reducing patient discomfort and extending diagnostic capabilities to settings where conventional systems or neurophysiologists are unavailable. However, certain factors should be considered when interpreting results and defining their clinical applicability.

The RETeval^®^ system, commercially launched in 2014, automatically adjusts flash strength according to pupil size. Response amplitudes and ITs can be influenced by factors such as pupil diameters larger than 6.5 mm, AL, and even sex [28,29]. In contrast, no significant changes have been observed in patients with smaller pupils, suggesting that RETeval^®^ performance is less affected in this subgroup. Nonetheless, compensating for the Stiles–Crawford effect would likely improve the accuracy of all recordings [29,31]. IT values may also vary in flicker ERG recordings at lower stimulus intensities, potentially due to the sequence of testing and dynamic changes in pupil diameter [31]. 

As a part of the implementation of portable ERG systems, several studies have focused on establishing normative values to account for methodological and technical differences compared with conventional systems [23,25,35]. Across studies, RETeval^®^ consistently produces lower amplitude values, typically ranging from 50% to 85% of those obtained with conventional systems [45], while IT values remain relatively consistent between systems [9,33,34]. Despite these amplitude discrepancies, RETeval^®^ has shown good intra- and inter-examiner reproducibility. ITs demonstrate higher reproducibility than amplitudes, which is an important consideration for diagnostic reliability [33,35]. Amplitude reproducibility is particularly variable for cone responses [9,23,33], although inter-examiner consistency has generally been reported as being moderate-to-high (ICC). While pupil size itself has not shown a significant correlation with flicker ERG responses, pupil parameters may still provide complementary information for distinguishing retinal function from post-retinal pathway abnormalities [23].

Device-related differences in ERG results have been attributed to factors such as electrode type, stimulators, and test conditions [73]. Skin electrodes, while improving patient comfort, are associated with lower amplitudes compared with corneal electrodes [32]. The extent of the retinal area stimulated by light also influences amplitude, particularly when comparing across recording systems [33]. Differences in stimulator design can produce variable ERG results [33,34]. To address these inconsistencies, the ISCEV has proposed separate normative datasets and calibration methods for different ERG platforms [74,75]. Despite technical variability, some studies have shown high diagnostic agreement between handheld and standard systems, with 100% sensitivity for detecting abnormal responses in selected cohorts [9,35]. 

Among its applications, the RETeval^®^ system has been most widely adopted for DR screening. Its high sensitivity [4,5,16] makes it a valuable tool for early detection, particularly in primary care and community-based settings. A major advantage is its patient- and operator-friendly design, which eliminates the need for mydriasis, corneal electrodes, or complex infrastructure. This simplicity, combined with portability, enables use by non-specialized healthcare personnel and supports mass screening programs for DR and VTDR. While the system substantially improves patient comfort and reduces examination time, a major limitation is its lower specificity, which contributes to a considerable rate of false positives and potentially unnecessary referrals [5,16].

The DR assessment protocol incorporates the IT, amplitude, age and pupil response parameters. While some studies have emphasized wave amplitudes as the most informative measure [5], others highlighted the greater diagnostic value of ITs [4]. Several investigations have focused on defining optimal cutoff values for the DR score [4,19]. Raising the cutoff to 22 has significantly improved RETeval^®^ performance, achieving 100% sensitivity and 92% specificity for detecting VTDR [4]. Excluding clinically significant DME enhanced diagnostic accuracy [16]. RETeval^®^ has also outperformed the OCT-derived metrics of macular RNFL thickness or CMT in detecting VTDR in DM patients without DME [4]. Importantly, its technical failure (1%) is much lower than that reported for retinal imaging systems (35%) [16,76]. Variability in DR scores between studies has been attributed to ethnicity, skin pigmentation, and glycemic control [19]. 

In DM patients, RETeval^®^ quantification of ITs and amplitudes in flicker ERG recordings with 16 and 32 Td·s stimuli has shown strong correlations with both DR severity and systemic metabolic status. This supports its role in detecting preclinical neurovascular dysfunction [17,18,19,20]. When combined with systemic and ocular risk factors (e.g., BCVA, diabetes control, disease duration), RETeval^®^ further improves diagnostic performance, with reported sensitivity and specificity of 93.3% and 80.3%, respectively. However, the device appears to be more effective in detecting advanced stages of DR, rather than in identifying early disease [20]. 

Despite these strengths, limitations remain. Under ISCEV-standardized conditions, RETeval^®^ and conventional ERG systems both detected functional changes under scotopic conditions, but conventional ERG performed better in identifying photopic changes [48]. Moreover, in long-standing DM1 patients without DR, RETeval^®^ did not detect abnormalities at 32 Td·s 28.3 Hz flicker or in DR risk scores. 

To further refine functional–structural correlations, several studies have combined RETeval^®^ with multimodal imaging, particularly for identifying diabetes-related microvascular disease or neurovascular changes in patients with peripheral retinal lesions [14,15,18,47]. These findings reinforce the potential value of functional testing as a complement to structural imaging, offering an additional dimension for risk stratification, especially for patients who are at risk of progression or those with peripheral pathology that is not captured by central retinal imaging.

Beyond DR, RETeval^®^ has also applied in other retinal and uveal diseases. In CRVO, some studies reported improvements in IT after anti-VEGF treatment [49], suggesting potential utility in monitoring ischemic recovery. However, others suggest that early IT changes may be attributable to the procedure itself, rather than the therapeutic response [50]. In non-ischemic CRVO, supernormal amplitudes detected with RETeval^®^ [51] have been associated with a milder ischemic profile and better prognosis, though evidence remains limited and requires further validation.

In Birdshot chorioretinopathy, RETeval^®^ flicker recordings showed good correlation with conventional ERG responses under standard protocols, supporting their role in cone system monitoring. However, in advanced cases, responses with handheld systems were often undetectable [55], limiting applicability in severe retinal dysfunction. The device has shown potential as a diagnostic tool in surgical contexts: in eyes with dense vitreous hemorrhage where fundus visualization was precluded, flicker ERG amplitudes recorded with RETeval^®^ accurately detected RRD [53]. Postoperatively, recordings remained feasible, even for partially gas-filled eyes, and early flicker responses correlated with visual recovery, highlighting its value as a complementary functional tool in select scenarios [54].

The device has also been investigated as a screening tool for inherited retinal dystrophies. While RETeval^®^ is effective in detecting severe cone dysfunction, its sensitivity in milder disease forms is limited, reducing its utility for screening in these conditions [52]. Discrepancies in waveform morphology and amplitude under photopic conditions when compared to the conventional tabletop ERG system due to a weaker stimulation of the OFF pathway may complicate the interpretation of results in complex retinal diseases and could lead to misdiagnosis [45]. 

The lack of evaluation in larger cohorts and across varying disease severities further underscores the need for more extensive, well-controlled studies to determine the extent to which RETeval^®^ can be reliably integrated into clinical workflows for diagnosing and monitoring retinal pathologies. Future research should prioritize refining recording protocols, optimizing stimulus parameters, and evaluating performance across a broader spectrum of both inherited and acquired retinal diseases.

The ability to record the PhNR as a functional marker of retinal ganglion cell activity in glaucoma, in a non-invasive and convenient manner, represents the primary rationale for introducing RETeval^®^ as a screening tool. Several parameters (such as P_72_, P_min_, W-ratio or BT) have been investigated using RETeval^®^. Among these, the PT/B ratio, which demonstrates a low CoR, has been identified as the optimal PhNR measure with RETeval^®^ [57].

One of the main challenges in integrating RETeval^®^ into glaucoma diagnostics lies in the variability introduced by the electrode type, recording protocols, and waveform processing methods. Studies have shown that DTL electrodes provide a superior signal-to-noise ratio and more precise PhNR measurements compared to sensor strips. Nevertheless, sensor strips can still yield reproducible and clinically acceptable PhNR recordings [57,59]. Methodological refinements, such as detrending techniques, have also been proposed to enhance repeatability and reduce variability, though they require careful application to preserve waveform morphology [58]. 

Although various RETeval^®^ PhNR parameters show strong correlations with structural (OCT) and functional (visual field MD) measures in moderate-to-advanced glaucoma, sensitivity in detecting early-stage glaucoma remains limited [60]. Certain PhNR metrics, such as the W-ratio and BT, have demonstrated changes in early glaucoma patients [11,61]. Moreover, RETeval^®^ analysis using artificial intelligence algorithms has shown higher classification accuracy than OCT in glaucoma, which is likely due to the integration of multiple parameters, though comparable performance was achieved with as few as two to four inputs [62]. 

Despite its limitations, RETeval^®^ appears to be most effective as a complementary tool for glaucoma monitoring, rather than as a primary diagnostic modality. It is particularly useful in moderate-to-advanced disease stages, where it helps to mitigate the floor effect seen in visual fields. However, it does not yet replace conventional structural or functional tests in the early detection setting. 

In pediatric settings, RETeval^®^ has emerged as a valuable alternative to traditional ERG, especially for infants, young children, or non-compliant patients who may not tolerate mydriasis, corneal electrodes, or lengthy procedures. Studies have demonstrated its utility for screening and functional assessment across a wide age range, including preterm neonates and patients under anesthesia. Its portability, non-mydriatic capability, and ease of use make it particularly attractive in settings where conventional ERG is impractical [9,13,66,69,70]. 

Flicker ERG, in particular, has shown promise as a diagnostic screening tool in children and has been evaluated in both healthy cohorts and pediatric patients with ocular disease [12,25]. RETeval^®^ flicker ERG has been used to establish age-specific normative data [25] and to assess patients with suspected cone dysfunction or nystagmus, demonstrating acceptable sensitivity and specificity [12]. Specific stimulation protocols have also been successfully applied in rare conditions such as BCM [67]. However, specificity remains limited, as false positives may occur due to fixation instability, eye movements, or inadequate pupil tracking, particularly in very young children or those with neurological comorbidities. Abnormal or ambiguous results therefore require confirmation with conventional ERG [12,35,66]. Moreover, normative datasets in pediatric populations remain sparse and demographically restricted, limiting their generalizability. Interpretation in infants requires age-specific considerations, as IT and amplitude evolve with retinal maturation and may be influenced by AL or refractive status [35,64,65,66].

A particular RETeval^®^ limitation in pediatrics is electrode placement. In small children and preterm infants, anatomical constraints can complicate optimal positioning, though this may be mitigated with smaller strip electrodes. Additional challenges include skin fragility, fixation stability, and frequent eye movement (e.g., in nystagmus), which introduce variability in amplitude and IT. These limitations often necessitate protocol adjustments, such as switching between Troland-based and fixed-candela protocols to account for differences in pupil size or unreliable pupil tracking [9,13,70]. 

Importantly, RETeval^®^ has been successfully used to record flicker ERG in sleeping or anesthetized infants, including preterm neonates, confirming its feasibility during surgical procedures. Optimal recordings have been obtained post-prandially, although higher intensity stimuli may pose difficulties [70,71]. RETeval^®^ also shows promise in monitoring cone dysfunction, assessing retinal function in premature infants, monitoring ROP, and detecting retinal toxicity in patients receiving vigabatrin therapy [13,66,69,71]. 

In children with RB, the Ephios handheld portable system has provided valuable functional assessments under anesthesia, revealing characteristic b-wave impairments and supporting its potential role in disease monitoring disease progression and treatment response [72]. 

Some reports, however, note that the device can be uncomfortable due to its close proximity to the face, and its sequential monocular testing protocol may be less time-efficient than conventional bilateral systems, potentially limiting its acceptability in a routine pediatric setting [9]. 

## 5. Conclusions

In conclusion, handheld ERG devices, particularly RETeval^®^, provide practical advantages, including portability, accessibility, non-mydriatic testing, and ease of use, which are especially valuable when conventional methods are impractical or when neurophysiology expertise is limited. They have demonstrated clinical utility in diverse settings, including pediatric populations, glaucoma, DR, and other retinal diseases. Their potential as a screening tool across multiple conditions, such as DR, VTDR, cone dysfunction and others, positions them as an important adjunct for functional retinal testing. Nonetheless, technical and interpretive limitations persist. Further research is needed to refine protocols, improve diagnostic accuracy, and better define their role in complex diseases. At present, handheld ERG systems should be considered complementary, rather than replacements for standard ERG. Integration into clinical practice requires cautious interpretation, as accurate analysis of ERG results depends on expertise in retinal physiology, stimulus-response dynamics, and artifact recognition. Without appropriate training, there is a risk of diagnostic inaccuracies, particularly in borderline cases; therefore, results should be interpreted by qualified professionals to ensure clinical validity and diagnostic accuracy.

## Figures and Tables

**Table 2:** Summary of studies assessing the diagnostic performance of the RETeval^®^ system in diabetic retinopathy (DR).

Author	Condition/Disease Patients/Controls	Device/Protocol	Main Findings	Limitations/Comments
Fukuo et al. [4]	118 DR (all grades)/48 controls	RETeval^®^, 28.3 Hz flicker (8 Td·s)	DR → ↑ IT (*p* < 0.001), ↓ amplitude (*p* < 0.01); IT correlates with severity (*r* = 0.55); AUC 0.84–0.89 (best for IT)	Small sample; limited stimulus range
Al-Otaibi et al. [5]	400 DR and VTDR screened for severity	RETeval^®^, flicker ERG (20 µV cutoff)	S 95.4%, SP 17.5%; 76% of FP had other fundus diseases; faster (5.3 min vs. 15 min) and preferred by patients (63% vs. 22.8%).	Low specificity; comorbidities not excluded; cutoff optimization needed
Weerasinghe et al. [14]	273 T2DM (high-risk population)	RETeval^®^ DR protocol + non-mydriatic fundus photography	DR S 73%, SP 70%; DR score ≥ 22 predictive; 59% previously undiagnosed	Moderate specificity; 15% failure rate (small pupils/poor fixation); cross-sectional; not suitable as standalone screening tool
Zhuang et al. [15]	97 T2DM (186 eyes) (94 with/92 without peripheral DR lesions)	RETeval^®^ (16 and 32 Td·s flicker) + UWF imaging + OCTA	Peripheral lesions → ↑ IT, ↓ amplitude, ↓ pupil ratio; UWF + ERG ↑ severity assessment	Moderate sample; variable lesion criteria; no follow-up
Maa et al. [16]	468 DR (various stages)	RETeval^®^, 28.3 Hz flicker (4, 8, 16, 32 Td·s)Undilated	AUC 0.86; S 83%, SP 78%; NPV 99%; ICC 0.90; test 2.3 min; failure 1% vs. 11% (fundus)	False positives; limited external validation
Değirmenci et al. [17]	42 DR (various stages, no DME)	RETeval^®^, DR assessment protocol + OCT	DR severity ↑ RETeval^®^ score; cutoff 22 → S/SP 92%; AUC 0.95 vs RNFL 0.56, 0.45 for IOP, and 0.65 for CMT	Small cohort; no DME; OCT not predictive
Zeng et al. [18]	T2DM (137 no DR/33 NPDR)	RETeval^®^, flicker ERG + OCTA	↓ Amplitude and VD even in no-DR; worsening with DR severity; IT delay correlated with age and VD loss	Cross-sectional; sex imbalance; possible OCTA artifacts
Zeng et al. [19]	172 T2DM (all stages, poorly controlled)	RETeval^®^ DR assessment protocol (16 and 32 Td·s, 28.3 Hz)	DR AUC 0.87 (S 80%, SP 82%); VTDR AUC 0.97 (S 95%, SP 89%); scores ↑ with severity	No controls; population-specific cutoff variation
Deng et al. [20]	232 T2DM (all DR stages)/70 controls	RETeval^®^ DR protocol (16 and 32 Td·s)	DR AUC 0.88 (cutoff 22.9); VTDR AUC 0.97 (cutoff 26.4); combining clinical data ↑ accuracy	Population-specific (Chinese); limited early DR sensitivity; cross-sectional; no longitudinal validation
Kirthi et al. [21]	29 prediabetes and 26 T2DM (55/20 controls)	RETeval^®^, flicker ERG (16 and 32 Td·s) + OCTA + CCM	↓ Amplitude and VD already in prediabetes; HbA1c ↑ → longer IT; 32 Td·s most sensitive	Small; cross-sectional; heterogeneous criteria
Zeng et al. [46]	66 T2DM no DR/62 controls	RETeval^®^, flicker ERG (16 and 32 Td·s) + OCT + OCTA	Delayed IT and ↓ amplitude correlated with ↓ vessel density and ↑ HbA1c → early neurovascular impairment	Cross-sectional; only T2DM; no causality established
Brigell et al. [47]	252 T2DM (4-year follow-up)	RETeval^®^ DR risk score + ETDRS grading	DR score ≥ 23.5 → 11× risk of intervention; ETDRS ≥ 53 → 3.5×; DME → 4.7×; combined structural + functional → 15× risk; each +1 score ↑ risk 1.28×	Single-center; no OCT confirmation of DME
Arias-Alvarez et al. [48]	23 T1DM without DR (46 eyes)/23 controls (46 eyes)	RETeval^®^ in Td·s vs. RETI-port/scan 21 in cd·s∙m^−2^ (ffERG + DR protocol)	Both systems detected early scotopic dysfunction; conventional ERG revealed photopic changes; DR protocol differences at 16 Td·s; strong inter-device correlation	Small sample; DR risk score less sensitive in early disease; photopic changes only in tabletop ERG

Abbreviations: AUC, area under the curve; BCVA, best-corrected visual acuity; CCM, corneal confocal microscopy; CMT, central macular thickness; DME, diabetic macular edema; DR, diabetic retinopathy; ERG, electroretinogram; ETDRS, Early Treatment Diabetic Retinopathy Study; ffERG, full-field electroretinogram; FP, false positives; HbA1c, glycated hemoglobin; ICC, intraclass correlation coefficient; IOP, intraocular pressure; IT, implicit time; NPDR, non-proliferative diabetic retinopathy; NPV, negative predictive value; OCT, optical coherence tomography; OCTA, optical coherence tomography angiography; RNFL, retinal nerve fiber layer; S, sensitivity; SP, specificity; T1DM, type 1 diabetes mellitus; T2DM, type 2 diabetes mellitus; Td·s, Troland-seconds; UWF, ultrawide-field; VD, vessel density; VTDR, vision-threatening diabetic retinopathy; and µV, microvolt; ↑ increase, ↓ decrease, → association or directional relationship between variables.

**Table 3:** Summary of studies assessing the clinical applications of the RETeval^®^ system in selected retinal and choroidal diseases.

Author	Condition/Disease Patients/Controls	Device/Protocol	Main Findings	Limitations/Comments
You et al. [45]	9 healthy subjects and 5 patients with CSNB or CPCPA	RETeval^®^ vs. Espion & LKC UTAS-E-3000; ISCEV protocols; DTL electrodes; dilated pupils	RETeval^®^ reproducible ERG responses but with ↓ photopic b-wave amplitudes (≈66–68% of Espion); faster IT; altered waveform morphology (slower return to baseline, absent i-wave, reduced photopic hill), reduced OFF-pathway (40b:20b ratio); greater discrepancies in CSNB	Very small sample; limited subtypes; amplitude/waveform discrepancies vs. tabletop ERG; reduced OFF-pathway stimulation; further validation needed before clinical equivalence
Yasuda et al. [49]	15 macular edema, secondary to CRVO (affected eye/fellow eye)	RETeval^®^ vs. UTAS visual system; 28.3 Hz flicker ERG	Prolonged IT in CRVO eyes; RETeval^®^ IT slightly longer but correlated with UTAS (*r* = 0.89); IT shortened after ranibizumab (32.2 → 30.6 ms, *p* < 0.001); amplitude unchanged → functional recovery	Small sample; reproducibility in ischemic diseases requires validation
Terauchi et al. [50]	79 AMD, DME, and RVO with macular edema	RETeval^®^, 28.3 Hz flicker ERG; baseline, <2 h, and 2–24 h post anti-VEGF (ranibizumab/aflibercept)	Flicker amplitude unchanged; IT prolonged post-injection in treated and fellow eyes; contralateral IT changes earlier with ranibizumab; suggests transient IOP/systemic effects	Small sample; possible bilateral disease confounding; early IT changes likely procedural/systemic, not therapeutic
Miyata et al. [51]	48 CRVO (ischemic and non-ischemic) (affected eye/fellow eye)	RETeval^®^, 28.3 Hz flicker ERG	IT prolonged in all CRVO; 20.8% showed supernormal amplitudes (≥117% fellow eye), linked to non-ischemic CRVO, mild IT delay, and better VA; amplitudes decreased after anti-VEGF only in supernormal group	Mechanistic explanation speculative; small sample; *p*-value near significance (*p* = 0.058); no long-term electrophysiologic follow-up
Nakamura et al. [52]	35 cone dysfunction syndromes/50 controls (33.1 ± 17.9 y [8–61])	RETeval^®^, 28.3 Hz flicker ERG (Td·s, no mydriasis; comparison with conventional ERG)	↓ 30 Hz flicker amplitudes and ↑ IT in patients; amplitude correlation between systems (*r* = 0.58, *p* < 0.001); no IT correlation (*r* = –0.07, *p* = 0.805); RETeval^®^ reliably detects severe cone dysfunction	IT not measurable in 14 eyes (low amplitude); flicker-only protocol; constant retinal illuminance sensitive to pupil size; variability limits mild case detection; standardized protocols needed
Han et al. [53]	69 vitreous hemorrhages ± RRD (affected eye/fellow eye)	RETeval^®^, 28.3 Hz flicker ERG	Flicker ERG recordable, despite media opacity; amplitude ratio ↓ in RRD; AUC 0.98 (S 100%, SP 95%); amplitude correlated with postoperative VA	Limited to dense hemorrhage; less reliable in PDR or macula-on RRD
Shibuya et al. [54]	17 rhegmatogenous retinal detachment after PPV	RETeval^®^, 30 Hz flicker ERG (pre- and post-PPV)	Flicker ERG measurable even with partial gas fill; early postoperative amplitudes predicted later recovery; greater improvement with ≤2 quadrants detached and early surgery (≤7 days)	Small sample; flicker-only; no macular assessment; short follow-up
Waldie et al. [55]	32 birdshot chorioretinopathy	RETeval^®^, non-mydriatic 28.3 Hz flicker (85 Td·s ± 32 Td·s protocols) vs. Espion conventional ERG	Strong correlation with conventional ERG (*r* > 0.75); minimal peak time differences; amplitudes ≈ 3× lower; amplitude ↓ with disease duration; undetectable responses in advanced cases	Lower amplitudes and waveform differences under photopic conditions; protocol choice affects interpretation; limited advanced-case sensitivity

Abbreviations: AMD, age-related macular degeneration; AUC, area under the curve; CPCPA, congenital post-receptoral cone pathway anomaly; CRVO, central retinal vein occlusion; CSNB, congenital stationary night blindness; DME, diabetic macular edema; ERG, electroretinogram; IOP, intraocular pressure; ISCEV, International Society for Clinical Electrophysiology of Vision; IT, implicit time; PDR, proliferative diabetic retinopathy; PPV, pars plana vitrectomy; RVO, retinal vein occlusion; RRD, rhegmatogenous retinal detachment; S, sensitivity; SP, specificity; Td·s, Troland-seconds; VA, visual acuity; VEGF, vascular endothelial growth factor.

**Table 5:** Summary of studies evaluating RETeval^®^ and other portable ERG systems in pediatric populations.

Author	Condition/Disease Patients/Controls; (Mean Age (Years), [Range])	Device/Protocol	Main Findings	Limitations/Comments
Carter et al. [9]	Healthy adults and pediatric patients with retinal dystrophies (37 children: 5 y [4 months–14 y]/44 adults: 39 y [19–75])	RETeval^®^ vs. Espion 300 (Diagnosys), ISCEV ffERG	RETeval^®^ amplitudes ↓ (except flicker ↑); IT similar; high diagnostic agreement (*κ* = 0.80); S 100%, SP 91%; detected all abnormal ERGs; test time 5–15 min vs. 3–10 min standard; practical for use by non-specialists	Five false positives; some recordings unfeasible (3 patients < 2 years); no absolute agreement; sensor strip issues, incomplete dark adaptation, and eye movements affected recordings; device- and age-specific normative data required; adults preferred tabletop (67%)
Grace et al. [12]	Children with nystagmus (34 with/31 without retinal dystrophy) and healthy controls (5.6 ± 2.7 y)	RETeval^®^, 30 Hz flicker ERG; dilated pupils	Successful recordings in 92% (65/71); nystagmus + dystrophy → ↓ amplitude, ↑ IT (*p* < 0.001); amplitude discriminated dystrophy vs. non-dystrophy (AUC = 0.986); cut-off < 5 µV → S 93%, SP 94.7%; <2.54 µV → S 93%, SP 100%; amplitudes ≥ 2.54 µV → 90% probability of normal cone function; good interocular agreement; age not influential	Eye-movement artifacts and fixation instability in nystagmus; no infants; interpret low amplitudes with caution
Ji et al. [13]	29 vigabatrin-treated children (<3 years) (13.6 months [6–27 months])	RETeval^®^, 30 Hz flicker ERG (non-sedated) vs. sedated ERG (Espion E2, Diagnosys LLC, Lowell, MA, USA)	Only 9/29 completed testing (agitation/movement issues); strong intra-visit reliability for amplitudes (ICC = 0.81–0.86), moderate for IT (ICC = 0.79–0.42); amplitudes correlated with Espion ERG (*ω^2^* = 0.71); smaller amplitudes but similar waveform	Low feasibility in very young children; motion/fixation artifacts; small cohort; electrode-related amplitude differences; reduced success without sedation
Soekamto et al. [64]	20 healthy children (38 eyes) [4–17 y]	RETeval^®^, dilated ffERG; ISCEV standard	Age correlated with OPs and DA 0.01 amplitudes (*r* = 0.59, *p* = 0.006) and with cone a-wave IT (*r* = 0.67, *p* = 0.001); no correlation with other ERG parameters; reliable ffERG without sedation	Small sample; cooperative patients only; demographic variability; no VA or refractive data; limited generalizability
Chan et al. [65]	479 healthy children5.0 ± 0.9 y [3.7–7.0]	RETeval^®^, non-mydriatic photopic ERG; SER +0.80 ± 1.00 D; AL 22.38 ± 0.70 mm	Age weakly correlated with ↑ b-wave and 30 Hz flicker amplitudes (*r* = 0.13–0.12, *p* < 0.01) and ↓ ITs (*r* = –0.13 to –0.18, *p* ≤ 0.01–0.001); AL correlated with several ERG parameters; findings reflect retinal maturation in preschool years	Eye-movement artifacts and electrode placement issues in very young children; non-mydriatic recordings reduce reliability; limited to Chinese cohort
Osigian et al. [66]	30 retinal dystrophies with/without general anesthesia) (4.2 y [10 months–18 y])	RETeval^®^, 30 Hz flicker ERG (pre- and post-anesthesia) vs. conventional sedated ffERG (E3 Diagnosys LLC)	RETeval^®^ amplitudes ↓ vs. conventional ERG pre- and post-anesthesia; strong amplitude correlations (*r* = 0.668–0.695, *p* < 0.001) and moderate IT correlations (*r* = 0.47–0.53, *p* = 0.090–0.027); 5 µV cut-off → PPV 85%, NPV 90%; suitable for cone dysfunction screening	Small sample; heterogeneous cohort; limited to 30 Hz cone flicker (misses rod dysfunction, e.g., CSNB); abnormal results need standard ERG confirmation
Haseoka et al. [67]	3 BCMmale patients (ages 6, 8, 12 y)	RETeval^®^, S-cone protocol; blue flashes (0.25–1.0 cd·s·m^−2^) on red background at 4.2 Hz; dilated pupils	Normal rod/max responses; markedly ↓ cone and flicker responses; positive S-cone responses at 30–40 ms with higher intensity (and both intensities in one case); confirms BCM diagnosis; feasible minimally invasive testing in children	Very small sample; no controls or normative S-cone data; limited to cone function assessment
Nagarajan et al. [68]	11 vigabatrin-treated children with infantile spasms (7.1 ± 2.9 months [3–16 months])	RETeval^®^, 30 Hz flicker ERG; baseline, 6 and 12 months after treatment	Flicker amplitudes ↓ after treatment (Δ 3.21 ± 2.45 µV at 6 m; 5.72 ± 4.18 µV at 12 m); 72.7% (8/11) showed retinal toxicity at 6 m; no recovery post-cessation; toxicity linked to treatment duration, not cumulative dose or age at onset	Very small sample; no controls; short follow-up; amplitude-only assessment; needs validation in larger cohorts
Tekavčič Pompe et al. [69]	25 preterm and 28 term-born children (with/without ROP) (6.9 ± 2.2 y/8.6 ± 1.9 y)	RETeval^®^, 30 Hz flicker ERG	T significantly prolonged in preterm children, especially with ROP (*p* < 0.0001): 25.76 ± 0.9 ms (controls), 26.87 ± 1.5 ms (preterm no ROP), 28.96 ± 1.0 ms (preterm ROP); amplitudes not significantly different; suggests delayed cone pathway maturation linked to OFF-bipolar cell development deficit	Small sample; cross-sectional design; moderate age difference; lower diagnostic sensitivity than Ganzfeld ERG
Hanson et al. [70]	28 term-born neonates (feasibility study for ROP-risk assessment)	RETeval^®^, 28.3 Hz flicker ERG; 3–50 cd·s∙m^−2^ stimuli through closed eyelids, no mydriasis	Reliable flicker ERGs in all infants; strongest responses at 30–50 cd·s∙m^−2^; measurable in 20/28 even at 3 cd·s∙m^−2^; amplitudes ↑ and ITs ↓ with stimulus strength; postprandial period optimal for testing; demonstrated feasibility for early cone assessment	Limited to term neonates; preliminary protocol; no comparison with preterm/ROP groups; lacks normative data
Taner et al. [71]	40 preterm-born children (with/without ROP)	RETeval^®^, 30 Hz flicker ERG through closed eyelids; stimuli 3–50 cd·s∙m^−2^	Most reproducible ERGs at 30 cd·s∙m^−2^; amplitudes ↑ with stimulus strength, ITs unchanged; recordings feasible in 88% of moderately and 85% of extremely preterm infants; higher amplitudes in extremely premature and ROP stage 2–3, suggesting accelerated maturation or ↑ light sensitivity	Small sample; few ROP cases; fragile skin and small facial structures hindered testing; sequential testing reduced success; lower reliability vs. term infants; improved electrodes and longitudinal studies needed
Sachidanandam et al. [72]	43 RB (48 eyes; 4.0 ± 2.4 y)/33 control eyes (4.3 ± 2.9 y)	Ephios hand-held ERG (Ephios AB, Teknikringen); under anesthesia; classified by IIRC	a- and b-wave amplitudes ↓ in all RB groups; significant b-wave loss in all except 30 Hz flicker; greater reduction in groups C–D vs. A–B; ITs ↑ in groups A–C; group D excluded (poor recordability), group E non-recordable; tumor extent > activity; one longitudinal case showed b-wave recovery during chemotherapy	Small cohort; possible anesthesia effects; limited IT data in advanced RB; single longitudinal case; no pediatric norms; amplitude loss may reflect bipolar cell dysfunction or treatment effect

Abbreviations: ERG, electroretinogram; ffERG, full-field electroretinogram; IT, implicit time; OPs, oscillatory potentials; DA, dark-adapted; S, sensitivity; SP, specificity; PPV, positive predictive value; NPV, negative predictive value; AUC, area under the curve; ICC, intra-class correlation coefficient; IIRC, International Intraocular Retinoblastoma Classification; CSNB, congenital stationary night blindness; ROP, retinopathy of prematurity; RB, retinoblastoma; BCM, blue cone monochromacy; AL, axial length; SER, spherical equivalent refraction; VA, visual acuity; and y, years.

## Data Availability

All data supporting the findings of this review are contained within the article. No additional datasets, analytic code, or data collection templates are publicly available.
